# Comparing the Clinical Outcomes Observed with Rivaroxaban Versus Warfarin for the Management of Obese Patients with Non-valvular Atrial Fibrillation: a Systematic Review and Meta-analysis

**DOI:** 10.1007/s10557-022-07361-9

**Published:** 2022-06-28

**Authors:** Xiaojun Zhuo, Jian Wang, Lihui Shao

**Affiliations:** Department of Cardiovascular Medicine, The Fourth Hospital of Changsha, Changsha, 410000 Hunan People’s Republic of China

**Keywords:** Rivaroxaban, Warfarin, Obesity, Atrial fibrillation, Stroke, Systemic embolism, Major bleeding

## Abstract

**Background:**

Atrial fibrillation (AF) is an irregular heart rhythm which is becoming more and more common in this new era. Obesity is a risk factor for cardiovascular events, and obese patients are more at risk for stroke. The Framingham Heart Study demonstrated an increase in the developmental risk of AF by 4% for every unit (kg/m^2^) increase in body mass index (BMI). An anticoagulant is often required for the management of such patients. In this analysis, we aimed to systematically compare the clinical outcomes which were associated with rivaroxaban versus warfarin for the treatment of obese patients with non-valvular AF.

**Methods:**

PubMed, EMBASE, Web of Science, http://www.ClinicalTrials.gov, Google Scholar, and Cochrane Central were the searched databases. Clinical outcomes including stroke, systemic embolism, and major bleeding were the endpoints. In this study, dichotomous data were analyzed by the RevMan software version 5.4. Risk ratio (RR) with 95% confidence interval (CI) was used for result interpretation.

**Results:**

Ten studies consisting of a total number of 168,081 obese participants were included whereby 81,332 participants were treated with rivaroxaban and 86,749 participants were treated with warfarin. The risks of ischemic (RR: 0.79, 95% CI: 0.74–0.84; *P* = 0.00001) and hemorrhagic stroke (RR: 0.61, 95% CI: 0.48–0.76; *P* = 0.0001) as well as systemic embolism (RR: 0.73, 95% CI: 0.62–0.87; *P* = 0.0004) were significantly lower with rivaroxaban compared to warfarin for the management of these obese patients with non-valvular AF. Rivaroxaban was also associated with a significantly lower risk of major bleeding (RR: 0.75, 95% CI: 0.65–0.87; *P* = 0.0001).

**Conclusion:**

Based on this analysis, rivaroxaban seemed to be a better option in comparison to warfarin, due to its association with significantly lower risks of stroke and bleeding outcomes in obese patients with non-valvular AF. However, this hypothesis should further be confirmed in larger clinical trials.

## Background

Atrial fibrillation (AF) is an irregular heart rhythm which is becoming more common in this new era. Several causes of AF have been identified including valvular heart diseases and non-valvular causes such as thyroid causes, hypertension, sleep apnea, exposure to cardiac stimulants, stress, or other idiopathic causes. Studies have shown that many patients with AF do not have a valvular heart disease [1]. Consequences of non-valvular AF include thromboembolic complications such as stroke and systemic embolism [2]. An anticoagulant is often required to manage patients with non-valvular AF [3].

Obesity is one among the most common risk factors associated with AF, and it should be noted that obesity can increase the prevalence of AF [4]. To confirm this statement, the Framingham Heart Study demonstrated an increase in the developmental risk of AF by 4% for every unit (kg/m^2^) increase in body mass index (BMI) [5]. Also, studies have shown an increase of 2- to threefold of AF in younger individuals with obesity, even though other risk factors are absent [6].

Anticoagulants are often required to prevent complications related to AF. For years, warfarin, a vitamin K synthesis inhibitor, has been used as an oral drug to prevent thromboembolic complications in patients with AF [7]. However, regular drug dosage adjustment was required to minimize the risk of bleeding or thrombosis based on the international normalized ratio (INR) value. Recently, several novel oral anticoagulants (NOACs), which are direct acting, and non-vitamin K antagonists, have been approved for use [8].

Due to limited data, previous studies have compared NOACs (all combined together) versus warfarin for the treatment of obese patients with non-valvular AF. There is seldom any meta-analysis that compared rivaroxaban versus warfarin in similar patients. In this analysis, we aimed to systematically compare the clinical outcomes which were associated with rivaroxaban versus warfarin for the treatment of obese patients with non-valvular AF.

## Methods

### Searched Databases and Searched Strategy

PubMed, EMBASE, Web of Science, http://www.ClinicalTrials.gov, Google Scholar, and Cochrane Central were the searched databases.

Studies that compared rivaroxaban versus warfarin for the treatment of obese patients with non-valvular AF were searched.

The following search terms or phrases were used:

- Rivaroxaban, warfarin, obese and atrial fibrillation;
- Rivaroxaban, warfarin, obesity and non-valvular atrial fibrillation;
- Novel oral anticoagulants, obesity, warfarin and atrial fibrillation;
- Non-vitamin K oral anticoagulants, obese, warfarin and atrial fibrillation.

### Inclusion and Exclusion Criteria

Inclusion criteria were:

Studies which compared rivaroxaban versus warfarin in obese patients with non-valvular AF;

Studies that reported clinical outcomes as their endpoints;

Studies that were published in English;

Studies that consisted of dichotomous data.

Exclusion criteria were:

Studies which were not based on obese patients with AF;

Studies that did not report clinical endpoints;

Studies that were published in a different language apart from English;

Studies that consisted of continuous data;

Duplicated studies.

### Data Extraction and Quality Assessment

The authors carefully and independently extracted data from each of the original studies including name of authors, year of publication, type of study, total number of obese participants who were treated with rivaroxaban and warfarin respectively, data representing the methodological quality of the studies, the baseline features of the participants, the body mass index, the clinical outcomes which were reported, the follow-up time period, and the number of events which were reported. Any disagreement which occurred during this data extraction process was carefully discussed among the authors and then a final decision was made by the corresponding author.

Quality assessment was carried out by the Newcastle Ottawa Scale (NOS) [9]. Based on this assessment, a grade was allotted to each study: grade “A” implying low risk of bias, grade “B” moderate risk whereas grade “C” implied high risk of bias.

### Outcomes and Definitions

The following outcomes were assessed:

- Ischemic stroke;
- Hemorrhagic stroke;
- Systemic embolism including deep vein thrombosis and pulmonary embolism;
- Gastrointestinal bleeding: defined as bleeding in the gastrointestinal tract;
- Any major bleeding. Please note that hemorrhagic stroke including intracerebral hemorrhage and subarachnoid hemorrhage was also included into the major bleeding category.

The endpoints which were reported in each of the original studies have been listed in Table 1.

**Table 1:** Outcomes which were reported in the original studies and the main features of the studies

Studies	Outcomes which were reported	Follow-up time period	Type of study	Bias risk grade	No. of obese participants who were treated with rivaroxaban (*n*)	No. of obese participants who were treated with warfarin (*n*)
**Alberts 2022 **[11]	Composite of stroke and systemic embolism, ischemic stroke, hemorrhagic stroke, systemic embolism, major bleeding	25 months	Retrospective cohort	B	21,547	21,547
**Berger 2021 **[12]	Stroke, ischemic stroke, hemorrhagic stroke, SE, major bleeding	12, 24, 36 months	Retrospective	B	10,555	5080
**Briasoulis 2021 **[13]	Ischemic stroke, gastrointestinal hemorrhage, hemorrhagic stroke, any major bleeding, all-cause mortality, myocardial infarction	19 months	Retrospective	B	4309	13,417
**Costa 2020 **[14]	Stroke and SE, ischemic stroke, major bleeding, intracranial hemorrhage, extracranial hemorrhage	2.3 years	Observational	B	1969	1969
**Deitelzweig 2020 **[15]	Stroke/SE, hemorrhagic stroke, ischemic stroke, SE, major bleeding, gastrointestinal bleeding, intracranial hemorrhage	8 months	Retrospective	B	29,146	30,902
**Kalani 2019 **[16]	Ischemic stroke, deep vein thrombosis, pulmonary embolism, myocardial infarction, major bleeding	-	Retrospective	B	33	90
**Kushnir 2019 **[17]	Stroke, major bleeding	-	Retrospective	B	174	152
**Perales 2019 **[18]	Venous thromboembolism, stroke, mortality, major bleeding	12 months	Retrospective	B	37	30
**Peterson 2019 **[19]	Ischemic stroke/SE, major bleeding	10 months	Observational	B	3563	3563
**Weir 2021 **[20]	Stroke/SE, ischemic stroke, hemorrhagic stroke, SE, major bleeding		Retrospective	B	9999	9999
**Total no. of obese participants (*n*)**					81,332	86,749

Abbreviations: *SE*, systemic embolism

### Statistical Analysis

In this study, dichotomous data were used. Statistical analysis was carried out by the latest version of the RevMan software, version 5.4. Heterogeneity was assessed by the *Q* statistic test and the *I*^2^ statistic test. A *P* value less or equal to 0.05 was considered statistically relevant. Heterogeneity increased with an increasing *I*^2^ value. The higher the percentage of *I*^2^, the higher the heterogeneity. If the *I*^2^ value was greater than 50%, a random effect statistical model was used, whereas a fixed effect statistical model was used when the *I*^2^ value was less than 50%.

Risk ratio (RR) with 95% confidence interval (CI) was used to represent the results following data analysis. Sensitivity analysis was also carried out to ensure that the result was not influenced by any of the studies. Publication bias was also assessed through funnel plots.

### Compliance with Ethical Guidelines

Ethical or board review approval was not required for this study. Data were extracted from previously published original studies, and no experiment was conducted by any of the authors.

## Results

### Searched Outcomes

The PRISMA guideline was followed [10]. A total number of 302 publications were searched from electronic databases. Following a careful assessment of the abstracts and titles, 254 publications were eliminated due to irrelevance. Forty-eight (48) full-text articles were assessed for eligibility. Further eliminations were carried out for the following reasons:

Systematic reviews and meta-analyses (2);

Did not report direct comparison of rivaroxaban versus warfarin (8);

Was not based on patients with AF (7);

Duplicated studies (21).

**Fig. 1:**
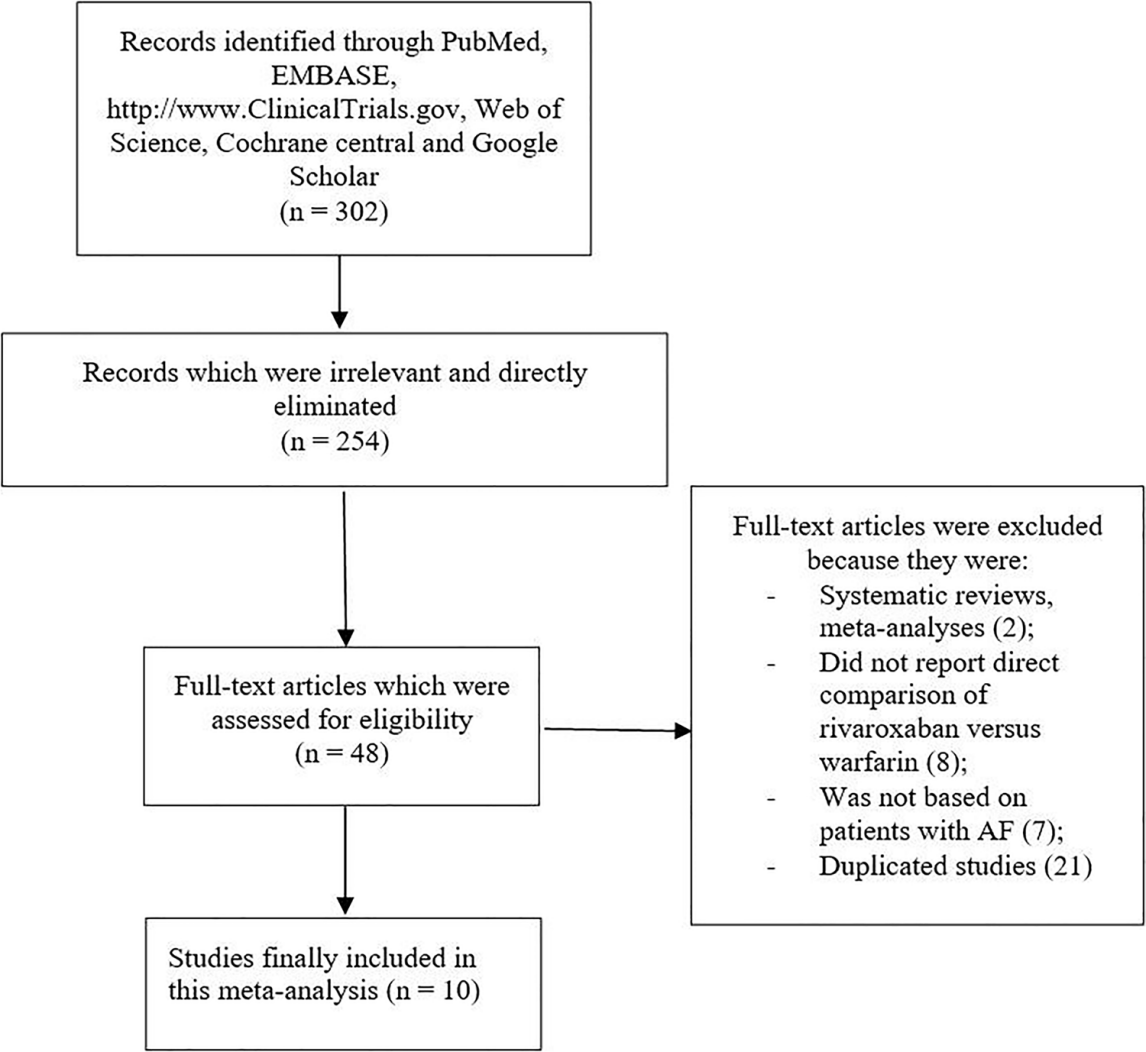
Flow diagram showing the selection of studies

Finally, only 10 studies [11–20] were selected to be used for this meta-analysis. The flow diagram for the study selection has been demonstrated in Fig. 1.

### Main Features of the Studies

Ten studies consisting of a total number of 168,081 obese participants were included in this analysis whereby 81,332 participants were treated with rivaroxaban and 86,749 participants were treated with warfarin as shown in Table 1. All the studies were observational studies. The study by Alberts (2022) and Deitelzweig (2020) consisted of the highest number of participants, whereas the study by Kalani (2019) and the study by Perales (2019) consisted of the lowest number of participants as shown in the table.

The BMI of the participants were reported in Table 2. It should be noted that all the participants were obese participants, but only a few studies categorized the BMI into obese, very obese, and extremely obese patients as shown in Table 2.

**Table 2:** Body mass index of the participants

Studies	BMI: 30–34.9 kg/m^2^	BMI: 35.0–39.9 kg/m^2^	BMI: ≥ 40 kg/m^2^
	Riv/War	Riv/War	Riv/War
**Alberts 2022**	50.7/50.7	14.7/14.7	34.6/34.7
**Berger 2021**	-	-	35.9/41.2
**Briasoulis 2021**	-	-	-
**Costa 2020**	39.3/38.6	26.4/26.8	34.4/34.7
**Deitelzweig 2020**	-	-	-
**Kalani 2019**	-	-	-
**Kushnir 2019**	-	-	-
**Perales 2019**	-	-	-
**Peterson 2019**	-	-	-
**Weir 2021**	41.4/43.1	14.7/14.3	43.8/42.6

Abbreviations: *BMI*, body mass index; *Riv*, rivaroxaban; *War*, warfarin

### Baseline Features of the Studies

The baseline characteristics of the participants are listed in Table 3. Male participants ranged on average from 36.7 to 90.0% with a mean age ranging from 55.0 to 72.3 years as shown in Table 3. Participants with diabetes mellitus (15.8% to 100%), hypertension (60.8% to 96.0%), coronary artery disease (16.2% to 54.6%), and current smokers (15.0% to 20.0%) are also listed in Table 3. In addition, the other medications used by the patients have been listed in Table 4.

**Table 3:** Baseline features of the participants

Studies	Age (years)	Males (%)	DM (%)	HBP (%)	CAD (%)	Smokers (%)
	Riv/War	Riv/War	Riv/War	Riv/War	Riv/War	Riv/War
**Alberts 2022**	65.1/65.3	64.1/63.9	15.8/16.8	85.5/83.8	16.2/18.6	-
**Berger 2021**	58.5/60.9	69.5/67.6	37.5/51.3	84.7/89.3	28.0/43.1	-
**Briasoulis 2021**	66.7/66.5	90.0/89.0	27.5/28.7	85.2/85.2	-	-
**Costa 2020**	-	50.7/49.7	45.1/46.4	86.2/85.3	-	15.1/16.1
**Deitelzweig 2020**	72.3/72.3	51.3/51.6	52.0/61.4	93.2/95.1	46.4/54.6	-
**Kalani 2019**	61.0/63.0	36.7/46.7	-	-	-	20.0/15.0
**Kushnir 2019**	60.9/66.8	45.0/41.0	-	-	-	-
**Perales 2019**	56.0/55.0	48.0/45.0	52.0/49.0	-	-	-
**Peterson 2019**	62.9/62.9	53.9/54.0	51.4/51.9	61.4/60.8	-	-
**Weir 2021**	70.0/70.2	58.8/58.0	100/100	96.0/95.8	34.0/34.6	-

Abbreviations: *DM*, diabetes mellitus; *HBP*, high blood pressure; *CAD*, coronary artery disease; *Riv*, rivaroxaban; *War*, warfarin

**Table 4:** Concomitant use of other medications in each subgroup of participants

Studies	Non-oral anticoagulants (%)	Antihyperlipidemics (%)	Antihypertensive agents (%)	Antiplatelet agents (%)
	Riv/War	Riv/War	Riv/War	Riv/War
**Alberts 2022**	11.5/12.1	9.10/10.1	92.2/92.5	12.1/12.5
**Berger 2021**	16.7/16.4	50.2/50.8	77.6/77.3	10.5/9.10
**Briasoulis 2021**	-	59.7/65.2	57.8/62.1	-
**Costa 2020**	21.4/21.8	52.4/55.9	72.6/72.7	50.3/52.0
**Deitelzweig 2020**	37.1/37.8	61.5/65.7	68.9/68.7	18.8/22.2
**Kalani 2019**	-	-	-	20.0/23.7
**Kushnir 2019**	-	-	-	-
**Perales 2019**	-	-	-	45.2/46.7
**Peterson 2019**	-	-	-	-
**Weir 2021**	14.8/15.4	72.1/72.2	92.3/92.0	16.1/16.7

Abbreviations: *Riv*, rivaroxaban subgroup; *War*, warfarin subgroup

### Main Results of This Analysis

**Fig. 2:**
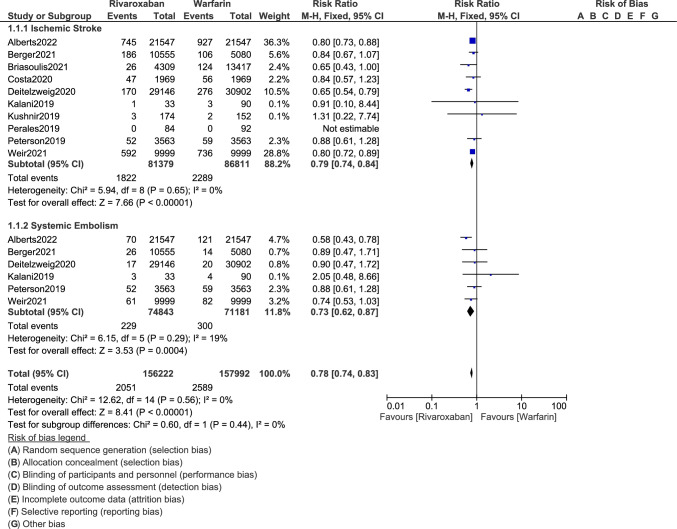
Comparing the clinical outcomes between rivaroxaban versus warfarin in obese patients with non-valvular atrial fibrillation (A)

**Fig. 3:**
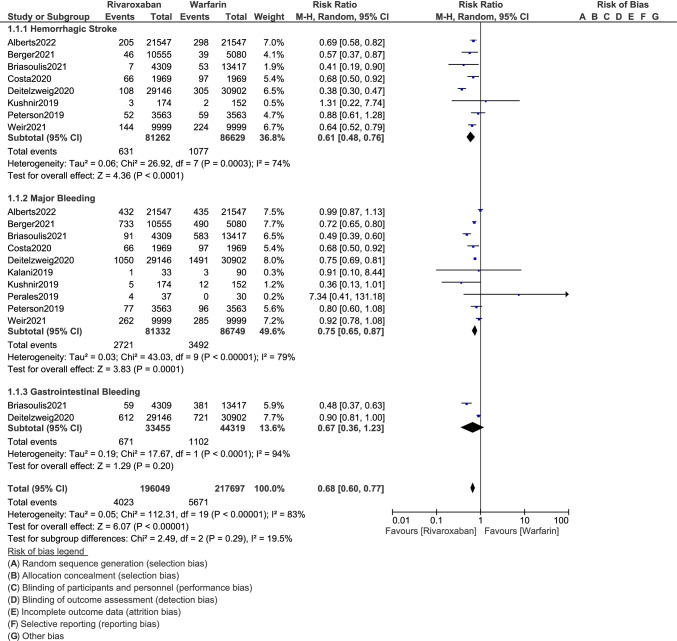
Comparing the clinical outcomes between rivaroxaban versus warfarin in obese patients with non-valvular atrial fibrillation (B)

The current results show that the risks of ischemic (RR: 0.79, 95% CI: 0.74–0.84; *P* = 0.00001) and hemorrhagic stroke (RR: 0.61, 95% CI: 0.48–0.76; *P* = 0.0001) were significantly lower with rivaroxaban as compared to warfarin for the management of these obese patients with non-valvular AF as shown in Figs. 2 and 3 respectively. The risk of systemic embolism (RR: 0.73, 95% CI: 0.62–0.87; *P* = 0.0004) was also significantly lower with rivaroxaban as shown in Fig. 2. Rivaroxaban was also associated with a significantly lower risk of major bleeding (RR: 0.75, 95% CI: 0.65–0.87; *P* = 0.0001) as shown in Fig. 3. However, the risk for gastrointestinal bleeding (RR: 0.67, 95% CI: 0.36–1.23; *P* = 0.20) was not significant.

The main results of this analysis have been summarized in Table 5.

**Table 5:** Main results of this analysis

Outcomes which were assessed	RR with 95% CI	P value	*I*^2^ value (%)
Ischemic stroke	0.79 [0.74–0.84]	0.00001	0
Hemorrhagic stroke	0.61 [0.48–0.76]	0.0001	74
Systemic embolism	0.73 [0.62–0.87]	0.0004	19
Major bleeding	0.75 [0.65–0.87]	0.0001	79
Gastrointestinal bleeding	0.67 [0.36–1.23]	0.20	94

Abbreviations: *RR*, risk ratios; *CI*, confidence intervals

Throughout this analysis, sensitivity analysis resulted in consistent results. Even though each study was excluded one by one by turn and a new analysis was carried out each time to observe for any significant change, no difference was observed in comparison with the main results of this analysis. For example, during the analysis for ischemic stroke, even if the study by Alberts et al. had a higher number of events and participants compared to most of the other studies, the final result was not influenced by this particular study. When the study by Alberts et al. was excluded from the analysis, and a new analysis was carried out for “ischemic stroke,” the results still showed ischemic stroke to be significantly lower with rivaroxaban (RR: 0.78, 95% CI: 0.72–0.84; *P* = 0.00001). Therefore, there was no impact of a dominant study on the final results. Results for the sensitivity analysis were consistent throughout.

**Fig. 4:**
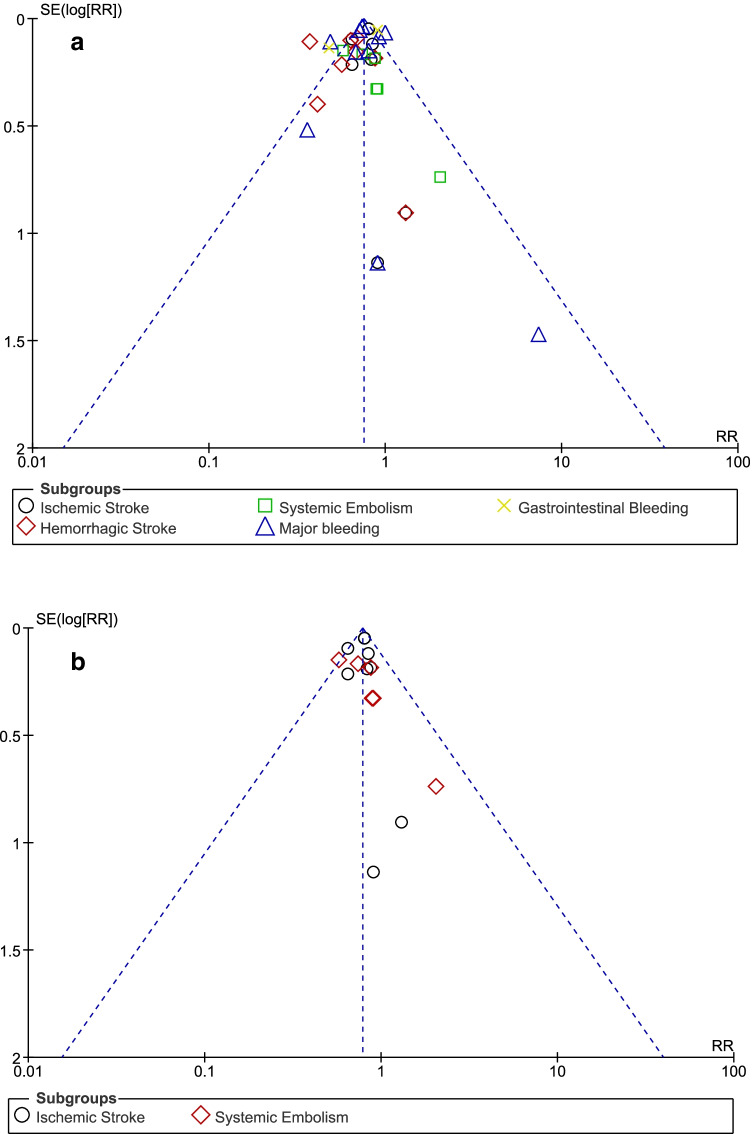
Funnel plots showing publication bias

Publication bias was visually assessed through funnel plots. Based on this visual assessment, there was only low evidence of publication bias across the studies which were included in this analysis. Publication bias was represented by Fig. 4.

## Discussion

In this analysis, a direct comparison of clinical outcomes was made with rivaroxaban versus warfarin in obese patients with non-valvular AF. Our results showed that rivaroxaban was associated with significantly lower risks of ischemic and hemorrhagic stroke, as well as a lower risk of systemic embolism. Major bleeding was also significantly less in comparison to warfarin.

Similarly, a meta-analysis which was published in the year 2021, comparing direct oral anticoagulants (DOACs) versus warfarin in 89,494 morbid obese patients with non-valvular AF showed DOACs to be effective and safe with statistical superiority in such patients, supporting the results of this current analysis [21]. Another recently published meta-analysis comparing the safety and efficacy of rivaroxaban and apixaban in patients with increased body mass showed positive outcomes [22]. However, the meta-analysis did not involve patients with AF.

Six years ago (in 2016), the International Society of Thrombosis and Haemostasis (ISTH) were against the use of DOACs in obese patients due to limited research on these types of participants [23]. However, due to more research based on the use of rivaroxaban in obese patients in recent years, it was proven that rivaroxaban and other DOACs had potential benefits compared to warfarin in obese patients, recommending its use.

In a retrospective, single-center cohort study based on obese patients with AF, the authors concluded that rivaroxaban might be considered an alternative to warfarin for such patients [24]. Apixaban also had similar positive response. However, the use of dabigatran in such a population required further confirmatory trials.

Nevertheless, even though rivaroxaban showed effective and safer results in comparison to warfarin for the treatment of obese patients with AF, the cost-effectiveness of this new anticoagulant should also be considered [25]. Hospitalization and outpatient visits have decreased with the use of rivaroxaban, due to a significantly lower bleeding risk, not requiring hospital visit or admission, compared to warfarin which is often associated with a higher INR value, with bleeding risks, requiring hospital admission for further management. Non-compliant to warfarin, or taking a larger amount of food rich in vitamin K could result in a low INR value, which will require frequent weekly visits for adjustment of dosage and regular blood tests to ensure INR value to be in the correct range. A study showed that the average total medical cost with rivaroxaban was $2829 lower compared to warfarin mainly because of hospital costs [26].

However, a few studies have also shown results which were different to this analysis. In a retrospective cohort study which was published last year, and which was based on the comparison of the efficacy and safety of anticoagulation between dabigatran (1290 participants) and rivaroxaban (1112 participants) in AF participants with different body mass index, the authors demonstrated that complications related to systemic embolism and stroke were higher in obese patients with higher BMI [27]. It was also shown that obese patients who were treated with rivaroxaban, and who had a higher BMI, were more at risk for earlier thrombosis. It was therefore suggested that the dosage of rivaroxaban should be increased in obese patients depending on their class of obesity; the higher the BMI, the higher the dosage of rivaroxaban, in order to minimize the risk of thrombotic complications. In our study, the different classes of obesity were not separately assessed due to lack of data, and hence we did not report any comparison of rivaroxaban use in different classes of obese patients. Nevertheless, another retrospective study [28] based on real-life cohort of 325 patients with AF who were treated with dabigatran, apixaban, and rivaroxaban, respectively, showed that obese patients with higher BMI, obesity class II and above, were at higher risks of stroke and bleeding depending on the anticoagulant drug subtype and the authors concluded that higher risk of bleeding was observed in the rivaroxaban subgroup, and this was different from the results of this current analysis.

Finally, this is one among the first meta-analysis assessing the direct comparison of rivaroxaban versus warfarin for the treatment of obese patients with non-valvular AF. The comparison of NOACs versus warfarin was previously made in obese patients with non-valvular AF. However, those studies compared a combination of NOACs including rivaroxaban, dabigatran, and apixaban versus warfarin. We needed a new study with a high number of participants which could compare rivaroxaban with warfarin for similar type of patients.

This study also has limitations. Outcomes such as myocardial infarction and all-cause mortality were not assessed since only very few studies reported those endpoints. The follow-up time periods were also not similar in all the studies. In addition, the intensity of obesity was not taken into consideration and therefore we could not carry out analysis based on the severity of obesity. Moreover, the cardiac medications and types of non-valvular AF were not taken into consideration and these factors could have an impact on the final results.

## Conclusions

Based on this analysis, rivaroxaban seemed to be a better option in comparison to warfarin, due to its association with significantly lower risks of stroke and bleeding outcomes in obese patients with non-valvular AF. In other words, rivaroxaban was more effective and safe in comparison to warfarin for similar patients. However, this hypothesis should further be confirmed in larger clinical trials.

## Data Availability

All data and materials used in this research are freely available in electronic databases (MEDLINE, EMBASE, http://www.ClinicalTrials.gov, Web of Science, Cochrane database, Google Scholar). References have been provided.

## Abbreviations

NOACs: Novel oral anticoagulants
AF: Atrial fibrillation
SE: Systemic embolism
RR: Risk ratio

## Declarations

### Authors’ Information

Xiaojun Zhuo is the first author of this manuscript.
